# AAV-NRF2 protects retinal and choroidal vasculature in a GDF15-dependent manner in an oxidative damage model of AMD

**DOI:** 10.1073/pnas.2616985123

**Published:** 2026-08-11

**Authors:** Shuai Wang, Sophia Zhao, Adam Daniels, Efrat Naaman, Apolonia Gardner, Lois E. H. Smith, Constance L. Cepko

**Affiliations:** ^a^Departments of Genetics and Ophthalmology, Harvard Medical School, Boston, MA 02115; ^b^HHMI, Chevy Chase, MD 20815; ^c^ Department of Ophthalmology, Boston Children’s Hospital, Harvard Medical School, Boston, MA 02115

**Keywords:** NRF2, GDF15, retinal vasculature, choriocapillaris, retinal pigment epithelium

## Abstract

Oxidative stress is proposed to be a driver of age-related diseases. Age-related macular degeneration (AMD) is one such disease, where the retinal pigment epithelium (RPE) is affected early in the disease. Vasculature damage also occurs, sometimes preceding RPE damage. To model some aspects of dry AMD, we used the NaIO_3_ mouse model of oxidative damage. Disruption of the deep retinal vascular plexus, disorganization and death of capillaries within the choriocapillaris, and marked electroretinographic decline were observed. Adeno-associated virus (AAV) overexpressing the transcription factor, NRF2, which induces antioxidation enzymes and represses inflammation, was tested for protection of damage. The BEST1 promoter limited expression to the RPE. The RPE, photoreceptors, and vascular architecture in both retinal and choroidal compartments were protected. Conditioned medium from RPE–choroid explants, infected by AAV8/BEST1-NRF2, was sufficient to transfer partial protection in vivo, suggesting that NRF2 induces a protective secreted factor(s). Analysis of RNA-seq data nominated growth differentiation factor 15 (GDF15) as a candidate secreted mediator. Injection of recombinant GDF15 reproduced key protective phenotypes in vivo, whereas Gdf15 deficiency attenuated NRF2-mediated rescue. Pharmacologic inhibition of TGF-β receptor signaling diminished NRF2-induced protection, supporting involvement of this signaling pathway. In a laser-induced choroidal neovascularization model, intravitreal GDF15 injection reduced fluorescein leakage and lesion size. These findings support a model in which NRF2 activation in the RPE induces expression of GDF15, which is capable of protecting the RPE, photoreceptors, and the retinal and choroidal vasculature. NRF2 and GDF15 have therapeutic potential for ocular diseases, as well as for other diseases with vascular pathology.

## Significance

Many diseases, particularly age-related diseases, are thought to involve oxidative stress. Vascular abnormalities occur in several of these diseases, including age-related macular degeneration (AMD). We are developing therapies for AMD and other vision-threatening diseases. Using an acute model of oxidative injury, we found disruption of two ocular vascular beds. These pathologies were prevented by an adeno-associated virus (AAV) vector expressing NRF2, a transcription factor that induces antioxidation genes and represses inflammation. NRF2 also induced the secreted factor growth differentiation factor 15 (GDF15), which was required for vascular protection. Recombinant GDF15 partially recapitulated the protective effects on the vasculature and other ocular cell types. These findings suggest GDF15’s therapeutic potential in ocular diseases and conditions involving stressed or damaged vasculature.

Age-related macular degeneration (AMD) is a leading cause of central vision loss in older adults. It is driven by a complex interplay of aging, genetic susceptibility, and environmental stressors (1, 2). Among the pathogenic mechanisms implicated in non-neovascular (dry) AMD, oxidative stress is thought to be a major driver, first affecting the retinal pigment epithelium (RPE) (3). Oxidative injury to this monolayer is considered a pivotal event in outer retinal failure as it supports the retina in several ways (3–5). It lies at the interface between the retina and the vasculature that supplies the outer retina, the choroid, forming the blood–retinal barrier. It regulates immune privilege, mediates the transport of nutrients and waste, and provides the critical chromophore for vision (4, 5). The RPE secretes vascular endothelial growth factor (VEGF), which is required for choroidal vascular development and maintenance, underscoring the importance of the RPE for this adjacent vascular bed (6, 7). Histopathologic studies of human AMD have further shown a close relationship between RPE abnormalities and choriocapillaris loss (8–12). Indeed, choriocapillaris dropout has been linked to drusen, accumulations of oxidized lipids and proteins that are a sign of AMD. In some analyses, choriocapillaris loss has been noted to precede other symptoms of AMD, supporting the view that vascular instability may be an early driver of AMD (9–11).

To investigate the relationship of the RPE, the choroid, and inner retinal vessels, an animal model of oxidative damage based upon injection of sodium iodate (NaIO_3_) is often used (13–16). Early toxicity to the RPE has been described, along with other features of outer retinal degeneration relevant to dry AMD (13). Although this model has traditionally been used to study RPE loss and photoreceptor damage, NaIO_3_ injury also perturbs the vasculature (13).

NRF2 is a master transcriptional regulator that positively regulates antioxidant and cytoprotective gene programs (17, 18). Impaired NRF2 signaling has been linked to increased vulnerability of the aging RPE, and genetic disruption of Nrf2 in mice leads to several phenotypes of AMD (19–21). Conversely, our previous work has shown that AAV-mediated overexpression of NRF2 in the RPE using the BEST1 promoter preserves RPE structure in a mouse model of an inherited retinal degeneration (22). In the acute NaIO_3_ model in mice and in rats, the same AAV8/BEST1-NRF2 prevented damage to the RPE and photoreceptors, and preserved vision (23). Whether NRF2 overexpression in the RPE can also stabilize neighboring retinal and choroidal vasculature has not been addressed.

One reason to suspect a protective role of NRF2 on the vasculature is that it not only regulates detoxification and redox homeostasis but also inflammatory and secretory programs (17, 18, 24). Among stress-induced secreted factors, growth differentiation factor 15 (GDF15) is of particular interest. GDF15 is a divergent TGF-β superfamily cytokine induced by mitochondrial stress, inflammation, and tissue injury, and has been recognized as a mediator of adaptation and tissue protection (25, 26). It is directly regulated by NRF2, among other transcriptional regulators. Although the canonical metabolic effects of circulating GDF15 are mediated through the hindbrain receptor, glial cell line-derived neurotrophic factor family receptor alpha-like (GFRAL) (27, 28), it has actions in peripheral tissues (25, 26). The mechanism of its action and the receptor(s) it uses more peripherally are unclear. In ocular systems, GDF15 is induced by retinal injury, can protect retinal ganglion cells, and has been implicated in limiting autoimmune uveitis through modulation of microglial responses (29–31). These observations make GDF15 a candidate for stress responsive signaling within the injured retina, but its potential role downstream of RPE-specific NRF2 overexpression, particularly in regulating ocular vascular stability, has not been examined. Here, we found that RPE-specific overexpression of NRF2 using AAV led to protection of choroidal and retinal vessels. The protection was partially dependent upon GDF15. Injection of recombinant GDF15 was found to recapitulate key aspects of NRF2-mediated protection, including preservation of retinal and choroidal vasculature, as well as RPE and photoreceptor structure.

## Results

### NaIO_3_ Induces Progressive Retinal and Choroidal Vascular Abnormalities with Functional Decline.

**Fig. 1.**
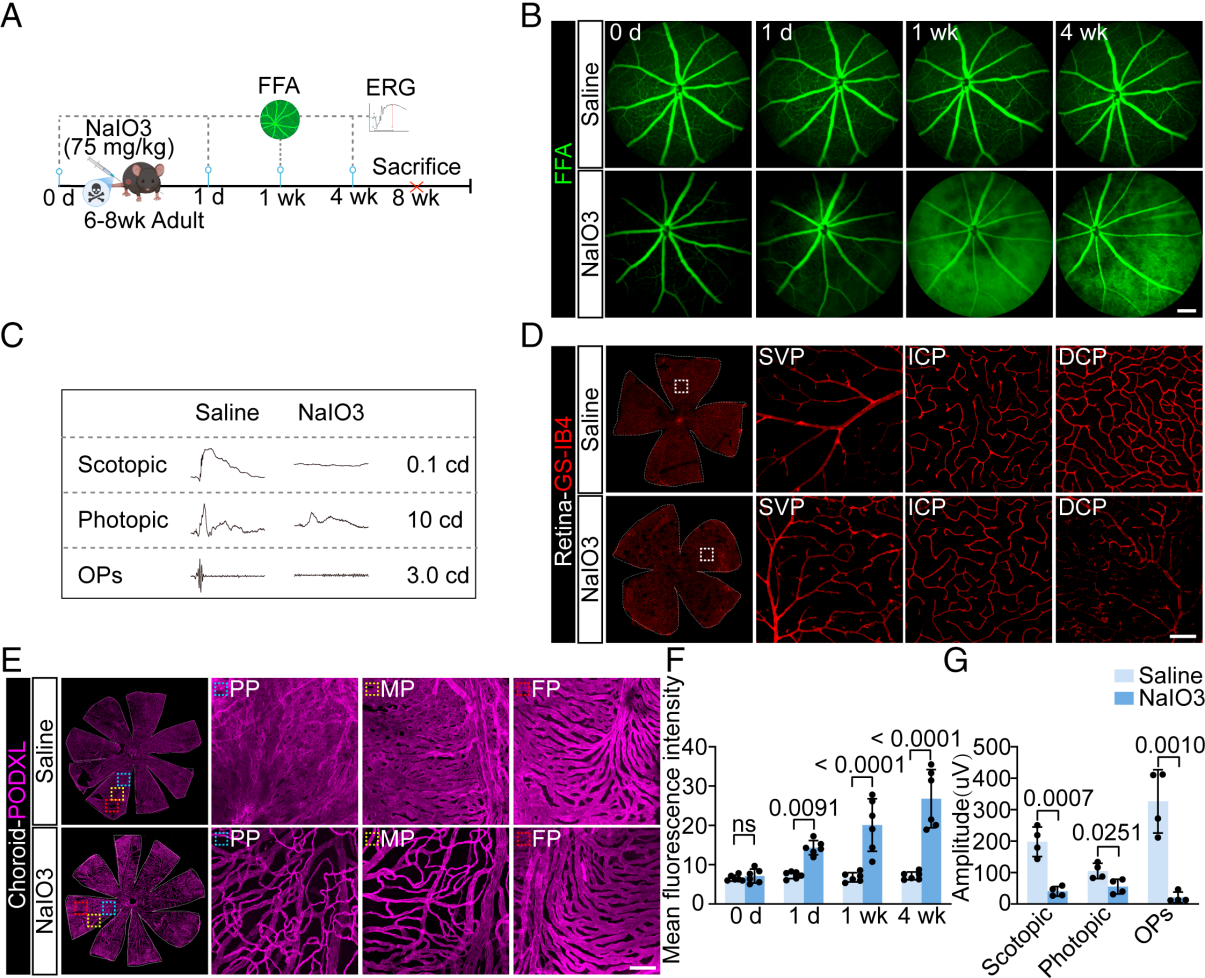
Characterization of retinal and choroidal changes following NaIO_3_ administration. (*A*) Experimental timeline illustrating systemic NaIO_3_ administration in adult mice. (*B*) Fundus fluorescein angiography (FFA) images at the indicated time points. (Scale bar, 100 μm.) (*C*) Representative electroretinography (ERG) recordings showing scotopic and photopic, oscillatory potentials (OPs) at 4 wk. (*D*) GS-IB4-stained retinal flat-mount. *Insets* show higher-magnification views of the superficial vascular plexus (SVP), intermediate capillary plexus (ICP), and deep capillary plexus (DCP). (Scale bar, 100 μm.) (*E*) Representative podocalyxin-stained RPE–choroid flat-mount images showing choroidal vasculature. Higher-magnification *Insets* indicate the posterior pole (PP, blue), mid-periphery (MP, yellow), and far periphery (FP, red). (Scale bar, 100 μm.) (*F*) Quantification of mean fluorescein intensity shown in *B* (n = 6 eyes per group, mean ± SD, two-way ANOVA with Šídák’s multiple-comparisons test). (*G*) Quantification of ERG waves shown in *C* (n = 4 eyes per group, mean ± SD, unpaired *t* test).

To define vascular and tissue changes induced by oxidative injury, adult mice were systemically administered NaIO_3_ and analyzed longitudinally by fundus fluorescein angiography (FFA), electroretinography (ERG), and flat-mount imaging of retinal and RPE–choroid tissues (Fig. 1*A*). Consistent with previous reports of NaIO_3_ injection (14), FFA revealed progressively increased hyperfluorescent signals over time, becoming more pronounced from 1 d to 1 wk, and further increasing by 4 wk after injury. Quantification of mean fluorescein signal intensity confirmed a significant treatment and time-dependent increase in the angiographic abnormalities in NaIO_3_-treated eyes (Fig. 1 *B* and *F*). Functional testing using ERG measurements demonstrated reduced scotopic and photopic responses, as well as impaired oscillatory potentials, at 4 wk in NaIO_3_-treated versus saline-treated controls (Fig. 1 *C* and *G*).

Retinal vessels were visualized in flat mounts using GS-IB4, a lectin commonly used to label endothelial cells and vascular networks. The SVP, ICP, and DCP were all well organized in control retinas. Following NaIO_3_ treatment, the SVP and ICP remained largely preserved, with no obvious alterations in overall vascular organization. In contrast, the DCP exhibited reproducible structural abnormalities, including irregular vascular trajectories and narrowed luminal profiles, indicating selective vulnerability of the DCP network (Fig. 1*D*). Because the RPE is immediately adjacent to the choriocapillaris, the choroidal vasculature also was examined. In control eyes, the choroidal vasculature exhibited distinct regional patterns: In the central posterior pole, the choriocapillaris formed a dense honeycomb-like meshwork; in the mid-peripheral region, the capillary network displayed a more linear arrangement with fewer interconnections and larger intercapillary spaces; and in the far periphery, choroidal vessels showed a characteristic palm-shaped pattern. Following NaIO_3_ treatment, these organized vascular patterns became disrupted (Fig. 1*E*). In the central posterior pole, the normally dense choriocapillaris meshwork became fragmented with reduced capillary density. In the mid-peripheral region, the vascular network lost its compact organization and appeared sparser and more atrophic, with enlarged intervascular spaces, while in the far periphery region there was little disruption. Together, these data define progressive angiographic abnormalities, retinal functional decline, DCP changes, and prominent choroidal vascular disruption, consistent with prior reports of NaIO_3_-induced angiographic changes and choriocapillaris atrophy (14–16).

### RPE-Specific NRF2 Overexpression Preserves Ocular Vasculature.

**Fig. 2.**
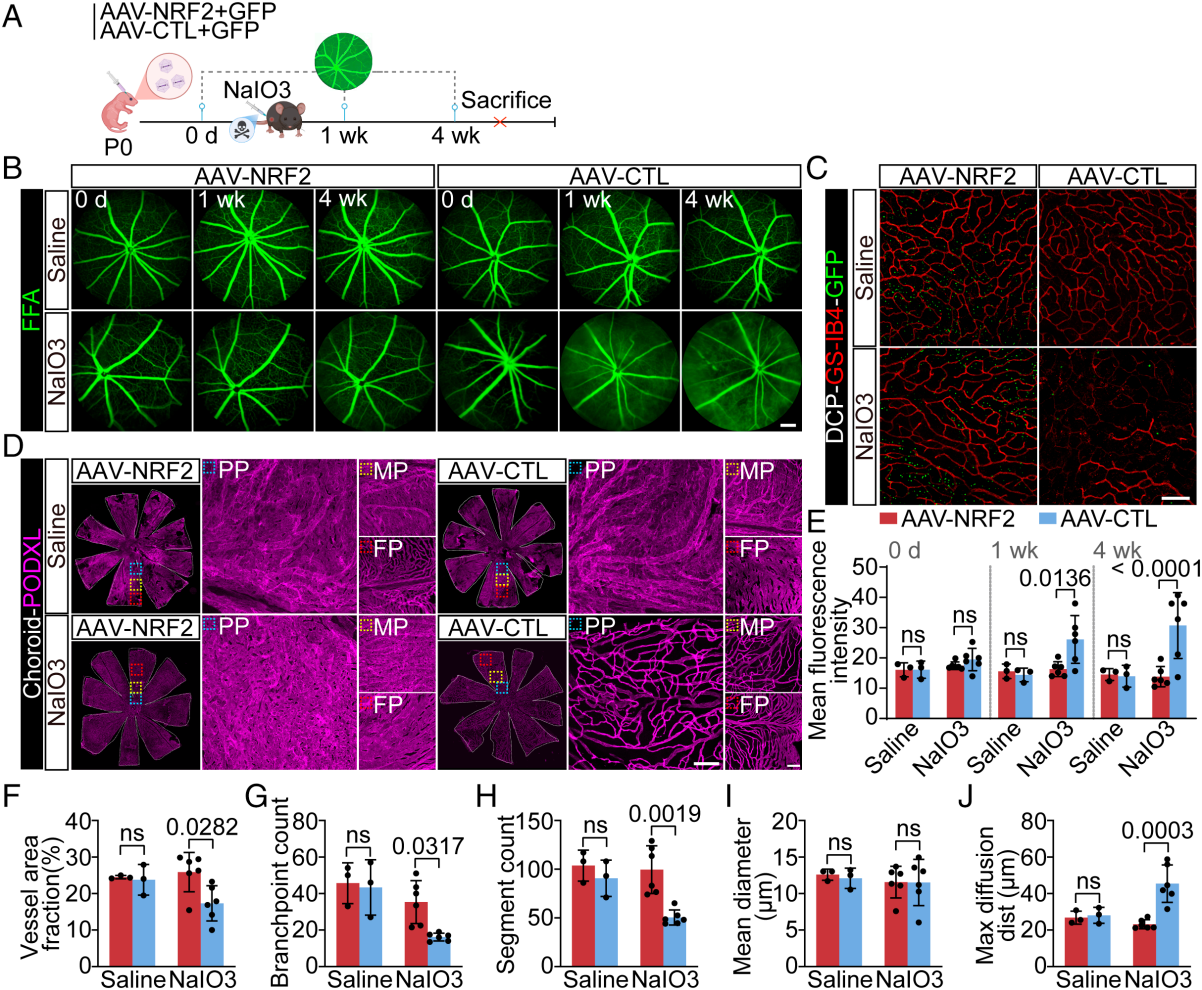
NRF2 expression in RPE preserves retinal and choroidal vasculature. (*A*) Experimental design: P0 subretinal delivery of AAV8/BEST1-NRF2 or AAV-CTL plus AAV-GFP tracer, followed by adult saline or NaIO_3_ administration. (*B*) FFA images after saline or NaIO_3_ administration. (Scale bar, 100 μm.) (*C*) GS-IB4-stained retinal flat-mount images showing the DCP. GFP indicates AAV-transduced retina. (Scale bar, 100 μm.) (*D*) Podocalyxin-stained RPE–choroid flat-mount showing PP (blue), MP (yellow), and FP (red). (Scale bar, 100 μm.) (*E*) Quantification of mean fluorescein intensity in *B*. (n = 3 eyes per group for saline and n = 6 eyes per group for NaIO_3_, mean ± SD, two-way ANOVA with Šídák’s multiple-comparisons test). (*F*–*J*) DCP vascular quantification in *C*. Same statistics as (*E*).

Given our previous study showing that NRF2 overexpression prevented damage to both RPE and photoreceptor cells following NaIO_3_ injection (23), it was of interest to determine if it could also preserve vascular architecture. To achieve RPE-specific expression, an AAV with the RPE-specific BEST1 promoter was used to drive NRF2 expression (22). As a control, an AAV using the same promoter but lacking a functional open reading frame (AAV8/BEST1-6xSTOPmutGFP, hereafter AAV-CTL) was used. These were delivered by subretinal injection at postnatal day 0 (P0), together with a small amount of a GFP vector (AAV8/RedO-H2B-GFP, hereafter AAV-GFP) to trace the infected area (23). Mice were allowed to mature, and vascular analyses were performed following NaIO_3_ administration. FFA revealed that eyes expressing AAV8/BEST1-NRF2 exhibited reduced angiographic abnormalities compared with AAV-CTL injected eyes after NaIO_3_ injection (Fig. 2 *B* and *E*). To further assess whether NRF2 could also protect the mature eye, AAV8/BEST1-NRF2 was injected subretinally into adult mice. NaIO_3_ was injected intraperitoneally, and FFA was performed. Because adult subretinal injection typically yields partial transduction, this paradigm provides localized, rather than global transduction (23). AAV8/BEST1-NRF2 reduced the hyperfluorescent signal in a partial and spatially restricted manner, with the effect most evident in GFP-positive regions (*SI Appendix*, Fig. S1). These data indicate that even when delivered to fully developed eyes, NRF2 is sufficient to confer localized vascular protection.

To evaluate retinal microvascular preservation, the DCP was examined in GS-IB4-stained retinal flat mounts. In AAV-CTL infected eyes, NaIO_3_ treatment caused disruption and simplification of DCP architecture. Eyes infected with AAV8/BEST1-NRF2 retained a denser and more continuous capillary network (Fig. 2*C*). GFP signal was detected broadly across the retina in these neonatal-injected samples, consistent with the extensive infection that is typical of neonatal injections. Quantitative analysis demonstrated that vascular area fraction, branchpoint, and segment number were higher in the AAV8/BEST1-NRF2 group, whereas maximal diffusion distance was lower compared with NaIO_3_-treated AAV-CTL infected eyes (Fig. 2 *F*–*J*). A lower maximal diffusion distance indicates fewer avascular spaces within the analyzed DCP field, consistent with improved vascular coverage and oxygen diffusion capacity. We also examined whether AAV8/BEST1-NRF2 infection preserved the choroidal vasculature. In AAV-CTL infected eyes in animals injected with NaIO_3_, the central posterior pole region showed marked loss and fragmentation of the normally dense choriocapillaris meshwork. In contrast, AAV8/BEST1-NRF2 eyes retained a more continuous vascular structure in this region. A similar pattern was observed in the mid-peripheral region, where AAV-CTL infected eyes displayed a looser vascular network with enlarged intercapillary spaces, whereas AAV8/BEST1-NRF2 infected eyes maintained a denser vascular organization (Fig. 2*D*). Together, these findings indicate that RPE targeted NRF2 overexpression preserves both retinal and choroidal vascular architecture following NaIO_3_-induced injury. The adult injection FFA data further indicate that NRF2 can provide vascular protection in mature eyes (*SI Appendix*, Fig. S1).

### AAV8/BEST1-NRF2-Induced Secreted Factor(s) Prevents Vascular Damage.

**Fig. 3.**
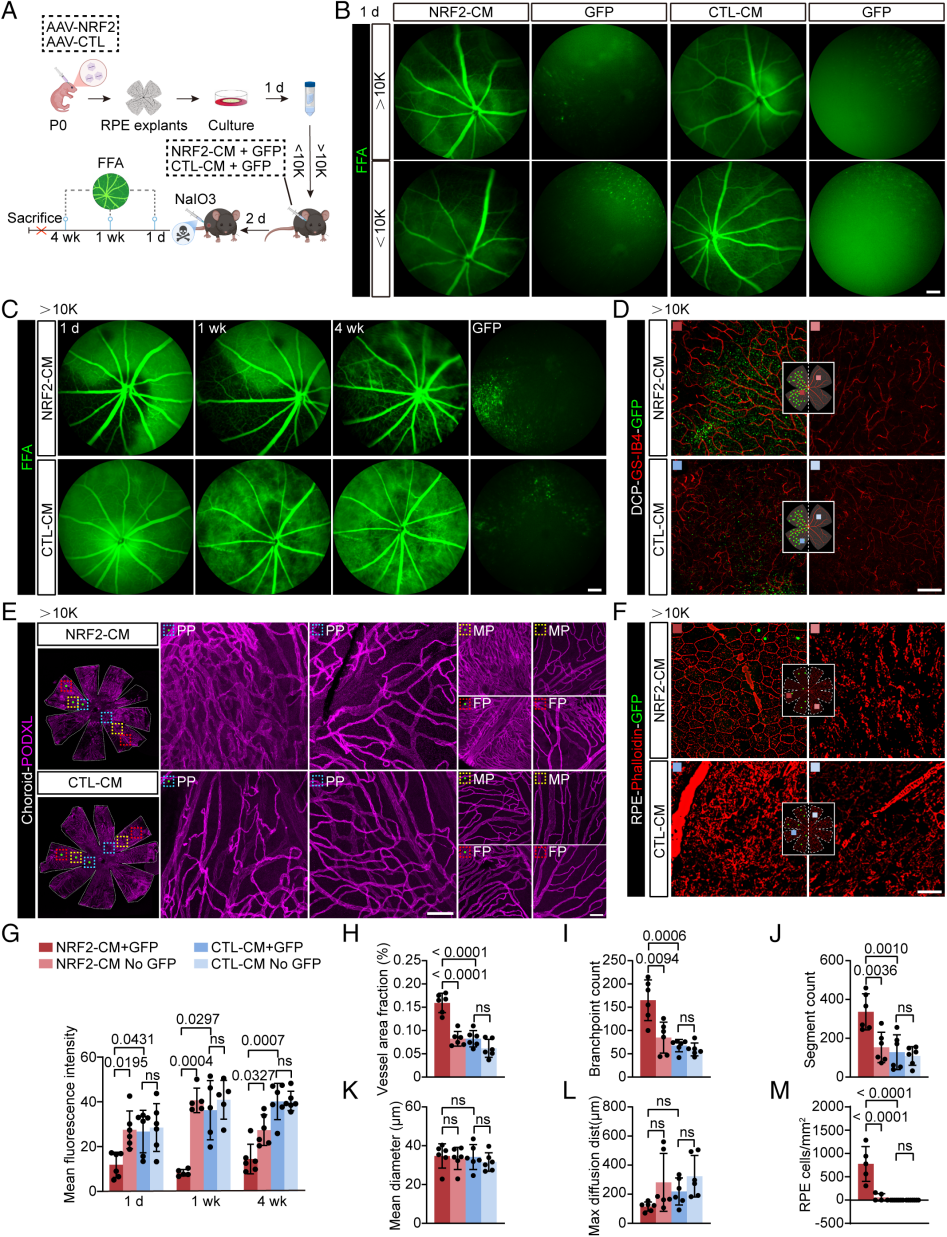
NRF2 conditioned medium transfers partial vascular protection in vivo. (*A*) Schematic illustration of conditioned medium (CM) preparation from RPE–choroid explants and in vivo transfer experiment. (*B*) FFA images 1 d after NaIO_3_ following subretinal injection of NRF2-CM or CTL-CM fractions (>10 kDa or <10 kDa) with GFP tracer. (*C*) Longitudinal FFA images following subretinal delivery of the >10 kDa NRF2-CM or CTL-CM with GFP tracer. (Scale bar, 100 μm.) (*D*) Retinal flat-mounts showing DCP. GFP marks the injected region. (Scale bar, 100 μm.) (*E*) Podocalyxin-stained RPE–choroid flat-mounts. Higher magnification views of choroidal vasculature in PP (blue), MP (yellow), and FP (red). (Scale bar, 100 μm.) (*F*) Phalloidin-stained RPE flat-mounts. (Scale bar, 50 μm.) (*G*) FFA quantification in *C* (n = 6 eyes per group, mean ± SD, two-way ANOVA with Tukey’s multiple-comparisons test). (*H*–*L*) Retinal vascular quantification in *D* (n = 6 eyes per group, mean ± SD, one-way ANOVA with Tukey’s multiple-comparisons correction). (*M*) RPE cell counts in *F* (n = 5 eyes per group for NRF2 and n = 7 eyes per group for CTL, mean ± SD, one-way ANOVA with Tukey’s multiple-comparisons correction).

To determine whether there was a secreted factor(s) produced by the RPE infected with AAV8/BEST1-NRF2 which confers vascular protection, conditioned medium (CM) was generated. RPE–choroid explants derived from mice infected with AAV8/BEST1-NRF2 or the AAV-CTL vector (NRF2-CM and CTL-CM, respectively) were used for this purpose (Fig. 3*A*). Explants were made from eyes infected in vivo at P0, and then aged to 8 wk. The explanted tissue was cultured for 24 h, and the collected supernatants were fractionated into >10 kDa and <10 kDa components using centrifugal filtration. Each fraction was delivered by subretinal injection into adult mice before NaIO_3_ administration, together with a GFP tracer virus. FFA performed 1 d after NaIO_3_ administration revealed that both the >10 kDa and <10 kDa fractions of NRF2-CM reduced hyperfluorescent signal compared with the corresponding CTL-CM fractions (Fig. 3*B*). The protective effect was localized to GFP-positive regions. Because both size fractions displayed protective activity, it was of interest to determine which size fraction had a higher concentration of protective activity. To this end, both size fractions of the NRF2-CM were diluted tenfold before subretinal delivery. The >10 kDa diluted material retained protective activity, whereas the <10 kDa fraction lost detectable protection, consistent with the idea that the protective activity is >10 kDa, likely a protein (*SI Appendix*, Fig. S2*A*).

Subsequent analyses were therefore conducted on the >10 kDa fraction. Longitudinal FFA showed that eyes receiving NRF2-CM exhibited markedly reduced diffuse hyperfluorescence at 1 d, 1 wk, and 4 wk after injury compared with eyes injected with CTL-CM (Fig. 3*C*). Quantification confirmed a significant reduction in fluorescein signal intensity in NRF2-CM-treated eyes (Fig. 3*G*). To directly examine retinal vascular architecture, retinal flat mounts were stained with GS-IB4. In GFP-positive regions, NRF2-CM-treated eyes displayed a more continuous and organized DCP than CTL-CM-treated eyes, whereas GFP-negative regions showed comparable vascular disruption between groups (Fig. 3*D*). Quantitative analysis of the DCP revealed increased vascular density, branchpoint number, and total vessel length, together with a reduction in maximal diffusion distance in NRF2-CM-treated retinas relative to CTL-CM controls. These observations demonstrated that NRF2-CM protects retinal vascular integrity (Fig. 3 *H*–*L*). The choroidal vasculature also was examined. In GFP-positive regions, NRF2-CM-treated eyes preserved a denser and more organized choriocapillaris network than CTL-CM treated eyes, although this preservation was less complete than that observed following infection with AAV8/BEST1-NRF2. In contrast, GFP-negative regions displayed similar vascular disruption in both groups (Fig. 3*E*). RPE morphology was assessed using phalloidin staining. Within GFP-positive regions, NRF2-CM-treated eyes showed partial preservation of the RPE cellular mosaic compared with CTL-CM-treated eyes, although the morphology was slightly less regular than that observed in uninjured tissue (Fig. 3 *F* and *M*). Together, these findings indicate that the RPE infected by AAV8/BEST1-NRF2 secretes at least one factor that is sufficient to confer partial, spatially restricted vascular protection against oxidative injury.

### Identification of Candidate Secreted Factors Downstream of NRF2.

**Fig. 4.**
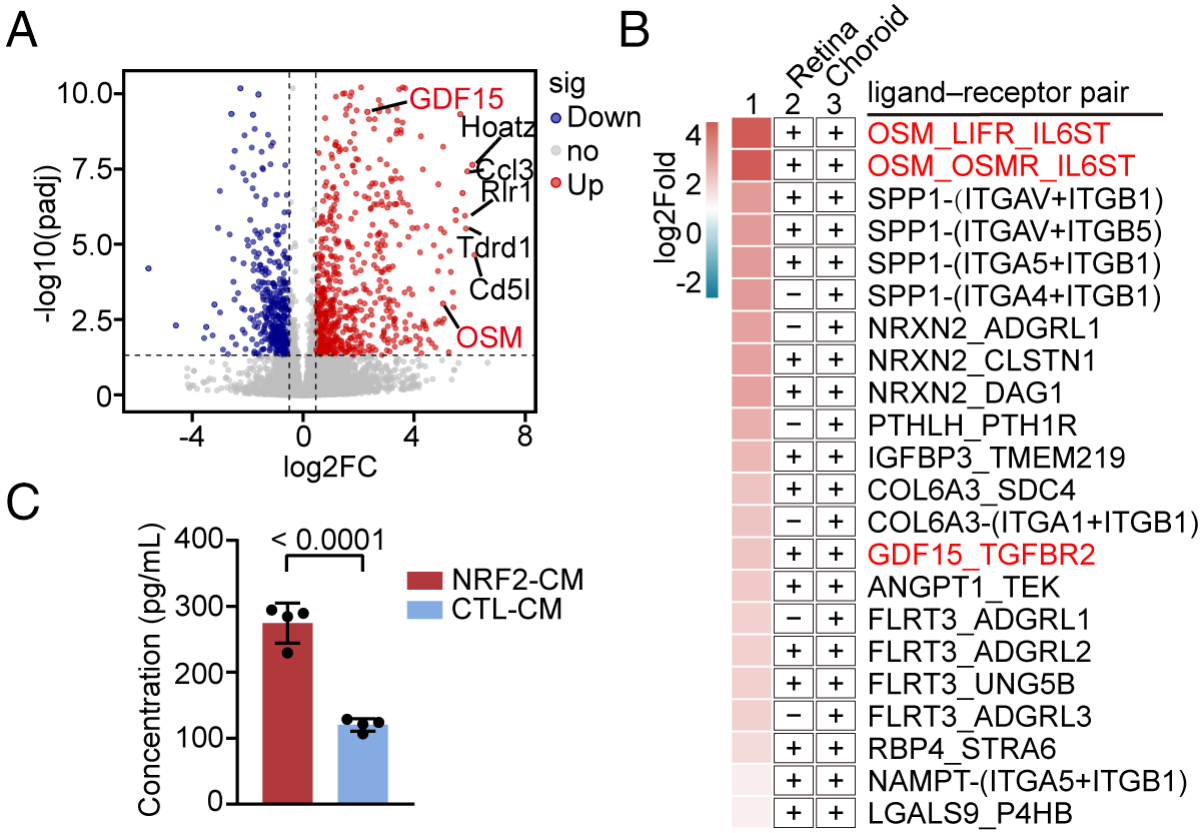
Identification of GDF15 as a candidate secreted effector induced by RPE NRF2 overexpression. (*A*) RNA-seq analysis of RPE samples from CD1 WT mice (22). Secreted factors upregulated in the RPE–choroid complex were identified after AAV8/BEST1-NRF2 infection relative to AAV-BEST1-GFP. (*B*) Ligand–receptor prioritization of NRF2-induced secreted ligands. Upregulated candidate secreted ligands from the RPE RNA-seq dataset were cross-referenced with receptor expression in retinal and choroidal endothelial cells from a non-neuronal ocular single-cell RNA-seq atlas (32). Each row shows a candidate ligand–receptor pair. The corresponding receptor was detected in retinal endothelial cells, choroidal endothelial cells, or both. Column 1 indicates the ligand log2 fold change in NRF2-expressing RPE cells from bulk RNA-seq data (22). Columns 2 and 3 indicate whether the corresponding receptor was detected in retinal or choroidal endothelial cells, respectively, based on single-cell RNA-seq data (32). Red text highlights the two candidate ligands selected for functional testing, OSM and GDF15. (*C*) ELISA quantification of GDF15 in unconcentrated NRF2-CM or CTL-CM (n = 4 eyes per group, mean ± SD, *t* test).

To identify candidate mediators of the vascular protective activity of the NRF2-CM, RNA-seq data generated from RPE infected by AAV8/BEST1-NRF2 (22) was analyzed for upregulated secreted factors. This dataset was collected from infection of CD1 wild-type (WT) animals that were not treated with NaIO_3_ (Fig. 4*A*). To prioritize candidates capable of signaling to vascular cells, upregulated genes were compared with a list of receptors that are in non-neuronal retinal cells, including vascular endothelial cells (32). By cross-referencing NRF2-induced secreted ligands with receptor expression across retinal and choroidal endothelial populations, we identified GDF15 and oncostatin M (OSM) as candidate mediators whose predicted receptors are expressed in vascular endothelial cells (Fig. 4*B*).

**Fig. 5.**
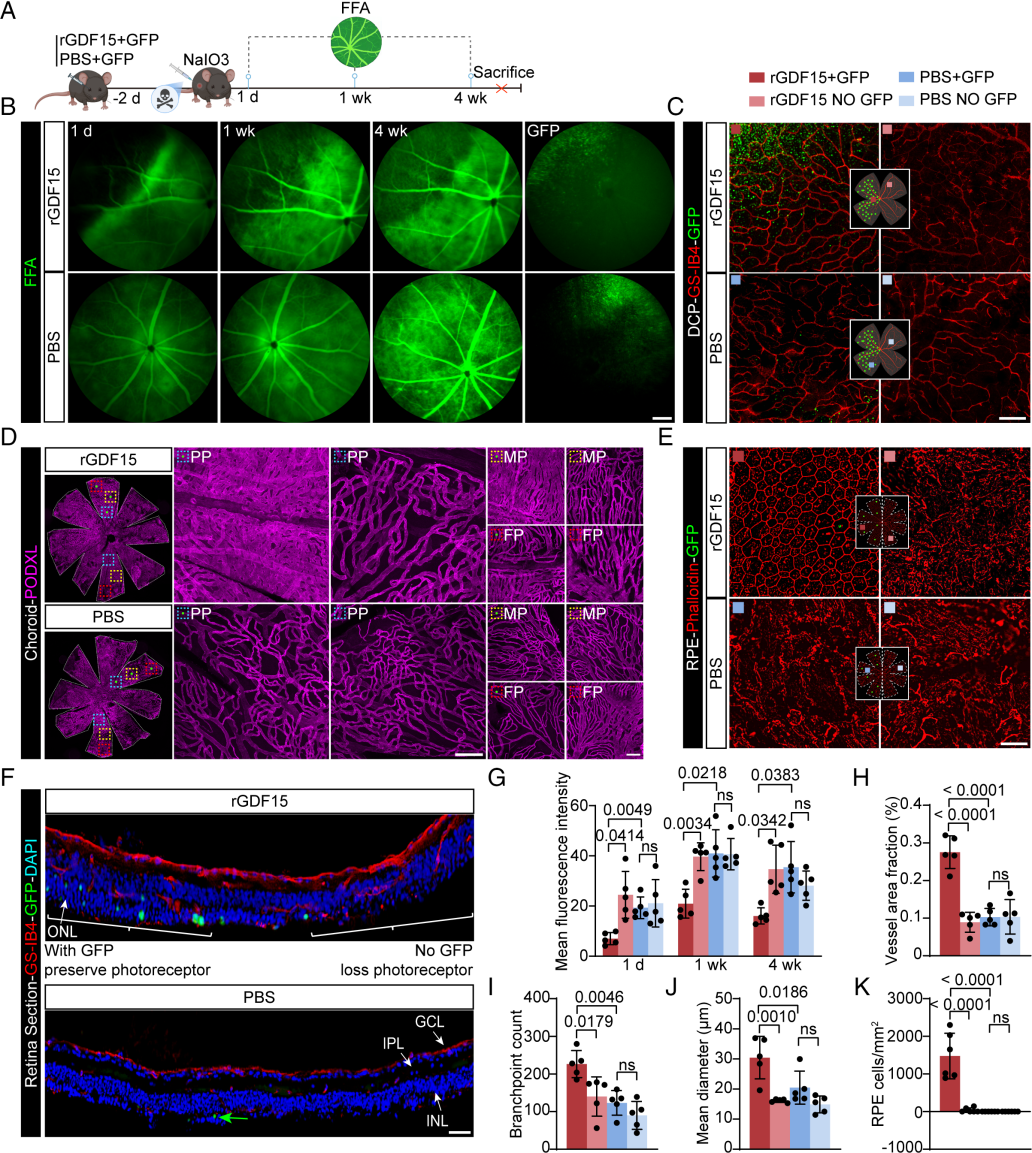
rGDF15 partially recapitulates NRF2-mediated vascular protection. (*A*) Experimental design: adult mice received a subretinal injection of recombinant mouse GDF15 (rGDF15, 10 μM, 1 μL/eye) or PBS with a GFP tracer. NaIO_3_ was administered 2 d later. (*B*) FFA images over time after NaIO_3_. GFP marks the injected region. (Scale bar, 100 μm.) (*C*) GS-IB4-stained retinal flat-mounts showing DCP. (Scale bar, 100 μm.) (*D*) Podocalyxin-stained choroidal vasculature in PP (blue), MP (yellow), and FP (red). (Scale bar, 100 μm.) (*E*) Phalloidin stained RPE flat-mounts. (Scale bar, 50 μm.) (*F*) Retinal sections showing photoreceptors in GFP-positive and GFP-negative regions. (Scale bar, 50 μm.) (*G*) Quantification of mean fluorescein intensity in *B* (n = 5 eyes per group, mean ± SD, two-way ANOVA with Tukey’s multiple-comparisons test). (*H*–*J*) Quantification of retinal vascular parameters in *C* (n = 5 eyes per group, mean ± SD, one-way ANOVA with Tukey’s multiple-comparisons correction). (*K*) Quantification of RPE cell counts in *E* (n = 6 eyes per group, mean ± SD, one-way ANOVA with Tukey’s multiple-comparisons correction).

We tested whether either factor could reproduce the vascular protective phenotype in vivo. Recombinant OSM or GDF15 was delivered subretinally before NaIO_3_ injury, and FFA analyses at 1 d and 1 wk post NaIO_3_ injury were performed. OSM showed no significant protection compared with PBS treated controls (*SI Appendix*, Fig. S2 *B* and *C*). However, GDF15 showed protection (Fig. 5 *B* and *G*). An enzyme-linked immunosorbent assay (ELISA) was carried out to determine if GDF15 was present in the CM, and if it was at a higher level in NRF2-CM relative to CTL-CM. Both were confirmed (Fig. 4*C*). Together, these findings identify GDF15 as induced by AAV8/BEST1-NRF2 and nominate it as a candidate mediator of NRF2-dependent protection.

### Recombinant GDF15 Partially Recapitulates AAV8/BEST1-NRF2-Induced Vascular and Tissue Protection.

To test whether GDF15 is sufficient to produce features of AAV8/BEST1-NRF2-induced protection, adult mice received subretinal injections of recombinant mouse GDF15 (rGDF15, 10 μM, 1 μL/eye) (31) or PBS, together with an AAV-GFP tracer, followed by NaIO_3_ administration 2 d later (Fig. 5*A*). Serial FFA showed that rGDF15 treated eyes exhibited reduced hyperfluorescent abnormalities compared with PBS treated eyes at 1 d, 1 wk, and 4 wk after injury (Fig. 5*B*). Comparison of GFP-positive and GFP-negative regions showed that the protective effect was localized to the injected area, indicating a local action of rGDF15 (Fig. 5*B*). To further assess retinal vascular structure, retinal flat mounts stained with GS-IB4 were analyzed. In the DCP, rGDF15 treated GFP-positive regions retained a more continuous capillary network, whereas PBS treated eyes displayed pronounced capillary disruption and vessel rarefaction following NaIO_3_ injury (Fig. 5*C*). Quantitative analyses demonstrated increased vessel area fraction, vessel length, branchpoint number, and segment count in rGDF15-treated retinas compared with PBS controls, along with reduced maximal diffusion distance (Fig. 5 *H*–*J* and *SI Appendix*, Fig. S3).

The choroidal vasculature was examined using podocalyxin immunohistochemistry on RPE–choroid flat mounts. In PBS-treated eyes, NaIO_3_ injury caused substantial disruption of the choriocapillaris network, with reduced capillary density and loss of the characteristic vascular architecture across central and peripheral regions. In contrast, rGDF15 treated eyes preserved a denser and more organized choriocapillaris network, particularly in the central posterior pole and mid-peripheral regions (Fig. 5*D*).

To determine if rGDF15 might also prevent RPE damage, RPE and photoreceptor morphologies were assessed by immunohistochemistry. In rGDF15 treated GFP-positive regions, the RPE monolayer retained a relatively intact hexagonal cellular mosaic. In contrast, PBS-treated retinas exhibited severe disruption of RPE organization, with irregular cell boundaries and loss of the typical epithelial structure. Adjacent GFP-negative regions in rGDF15-treated eyes similarly showed marked RPE degeneration following NaIO_3_ injury (Fig. 5 *E* and *K*). Retinal sections revealed that photoreceptor structure and number were better preserved in rGDF15 injected GFP-positive regions. In PBS-treated eyes, NaIO_3_ injury resulted in substantial thinning of the outer nuclear layer reflecting loss of photoreceptor cells. In contrast, rGDF15 treated regions maintained a thicker photoreceptor layer, whereas GFP-negative regions showed photoreceptor degeneration similar to PBS controls (Fig. 5*F*).

To explore the effective dose range, mouse rGDF15 was delivered subretinally at 50, 10, or 1 ng per eye before NaIO_3_ injury. The 50 ng dose reduced FFA hyperfluorescence at 1 d after injury, whereas 10 and 1 ng did not show clear protection at this early time point (*SI Appendix*, Fig. S4). By 1 wk, however, protection became detectable in the lower-dose groups, indicating that rGDF15-mediated protection can occur at lower doses but with delayed onset.

Together, these results show that mouse rGDF15 reduces NaIO_3_-induced angiographic abnormalities of both the retinal and choroidal vasculature. In addition, rGDF15-treated regions showed better preservation of RPE cells and photoreceptors.

### Loss of GDF15 Partially Attenuates NRF2-Mediated Functional and Vascular Protection.

**Fig. 6.**
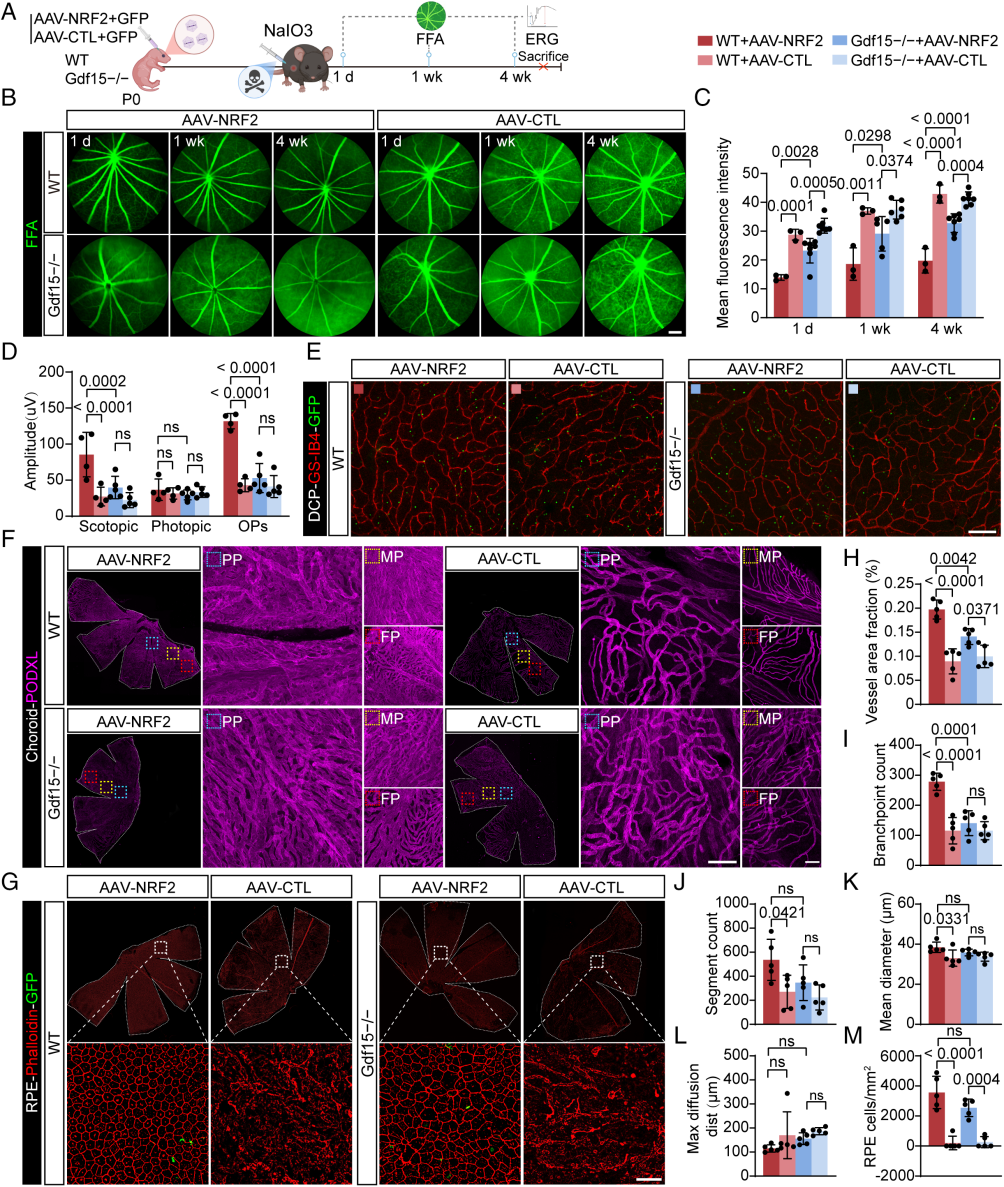
Loss of GDF15 attenuates NRF2-mediated protection following NaIO_3_-induced injury. (*A*) Experimental design: WT and Gdf15^–/–^mice received neonatal subretinal injection of AAV8/BEST1-NRF2 or AAV-CTL followed by adult NaIO_3_ injury and analysis. (*B*) FFA images after NaIO_3_. (Scale bar, 100 μm.) (*C*) Quantification of fluorescein intensity in *B* (n = 3 eyes per group for WT, n =7 eyes per group for Gdf15^–/–^mice, mean ± SD, two-way ANOVA with Tukey’s multiple-comparisons test). (*D*) Quantification of ERG responses in *SI Appendix*, Fig. S5*B* (n = 4 eyes per group for WT, n = 5 eyes per group for Gdf15^–/–^, mean ± SD, two-way ANOVA with Tukey’s multiple-comparisons test). (*E*) GS-IB4-stained retinal flat-mounts showing DCP. (Scale bar, 100 μm.) (*F*) Podocalyxin-stained RPE–choroid flat-mounts showing choriocapillaris morphology in PP (blue), MP (yellow), and FP (red). (Scale bar, 100 μm.) (*G*) Phalloidin-stained RPE flat-mounts. (Scale bar, 50 μm.) (*H*–*L*) Quantification of retinal vascular parameters in *E* (n = 5 eyes per group, mean ± SD, one-way ANOVA with Tukey’s multiple-comparisons correction). (*M*) RPE cell counts in *G* (n = 5 eyes per group, mean ± SD, one-way ANOVA with Tukey’s multiple-comparisons correction).

The rGDF15 injections demonstrated that rGDF15 was sufficient to produce several of the protective features induced by AAV8/BEST1-NRF2 after NaIO_3_ injury. To determine whether GDF15 is necessary for the protective effects of AAV8/BEST1-NRF2, we examined Gdf15^−/^^−^mice infected by AAV8/BEST1-NRF2 and subjected to NaIO_3_ injection (Fig. 6*A*). FFA showed that the reduction in hyperfluorescence was diminished in Gdf15^–/–^mice (Fig. 6 *B* and *C*). To determine if loss of GDF15 might affect retinal physiology, the Gdf15^–/–^mice, without AAV infection or NaIO_3_ injection, were tested by ERG. Baseline ERG responses were comparable between WT and Gdf15^–/–^mice across scotopic, photopic, and oscillatory potential measurements (*SI Appendix*, Fig. S5*A*). We therefore examined whether loss of GDF15 altered the functional benefit conferred by AAV8/BEST1-NRF2 after NaIO_3_ injury. ERG analysis showed that infection by AAV8/BEST1-NRF2 partially preserved retinal function in WT animals following NaIO_3_ treatment, as we previously reported (23) (Fig. 6*D* and *SI Appendix*, Fig. S5*B*). Under scotopic conditions, AAV8/BEST1-NRF2 infected WT eyes exhibited increased b-wave amplitudes compared with control eyes. In contrast, this improvement was significantly attenuated in Gdf15^–/–^mice, in which AAV8/BEST1-NRF2 infection produced only a modest and nonsignificant increase in scotopic responses. Photopic b-wave responses were not significantly altered by AAV8/BEST1-NRF2 infection in either genotype, indicating limited effects on cone mediated responses under these conditions. In contrast, oscillatory potentials, which reflect inner retinal activity, showed the most pronounced AAV8/BEST1-NRF2-dependent improvement, as well as dependence on Gdf15 after AAV8/BEST1-NRF2 infection. As Gdf15 has been reported to be expressed by, and protective of, retinal ganglion cells, the oscillatory potential protection may reflect an effect on this inner retinal cell type. Alternatively, or additionally, protection may be due to vascular preservation and/or direct effects on other inner retinal cell types.

To examine retinal vascular architecture, retinal flat mounts were analyzed. In WT retinas, AAV8/BEST1-NRF2 infection preserved a more continuous capillary network in the DCP after injury. In contrast, Gdf15-deficient retinas exhibited greater disruption of capillary organization and reduced vascular integrity despite AAV8/BEST1-NRF2 infection (Fig. 6 *E* and *H*–*L*). The choroidal vasculature was assessed using podocalyxin stained RPE–choroid flat mounts. In WT mice, AAV8/BEST1-NRF2 infection preserved a denser and more organized choriocapillaris network following NaIO_3_ injury. This choroidal protection was weakened in Gdf15^–/–^animals, which exhibited lower capillary density and more pronounced vascular disorganization across posterior and peripheral regions despite NRF2 expression (Fig. 6*F*). Thus, GDF15 also contributes to the ability of AAV8/BEST1-NRF2 infection to preserve choroidal vascular structure under oxidative stress.

RPE morphology was evaluated by phalloidin staining to determine if GDF15 was required for AAV8/BEST1-NRF2 mediated protection following NaIO_3_ injury (Fig. 6 *G* and *M*). In WT mice, AAV8/BEST1-NRF2 infection preserved the hexagonal RPE cellular mosaic after NaIO_3_ injury. In Gdf15^–/–^mice, the RPE monolayer remained relatively preserved, although mild irregularities in cell boundaries were occasionally observed. Consistent with this observation of the RPE, cone counts were preserved by AAV8/BEST1-NRF2 in both WT and Gdf15^–/–^mice, with no significant difference between NRF2-treated WT and Gdf15^–/–^groups (*SI Appendix*, Fig. S6). These findings suggest that GDF15 contributes only modestly to the protective effects of NRF2 on the RPE and that other mechanisms are likely sufficient to protect the RPE from NaIO_3_ injury. Together, these findings indicate that loss of GDF15 partially attenuates NRF2-mediated protection of retinal function and vascular integrity.

### Pharmacological Inhibition of TGF-β Receptor Signaling Attenuates NRF2 Mediated Protection.

**Fig. 7.**
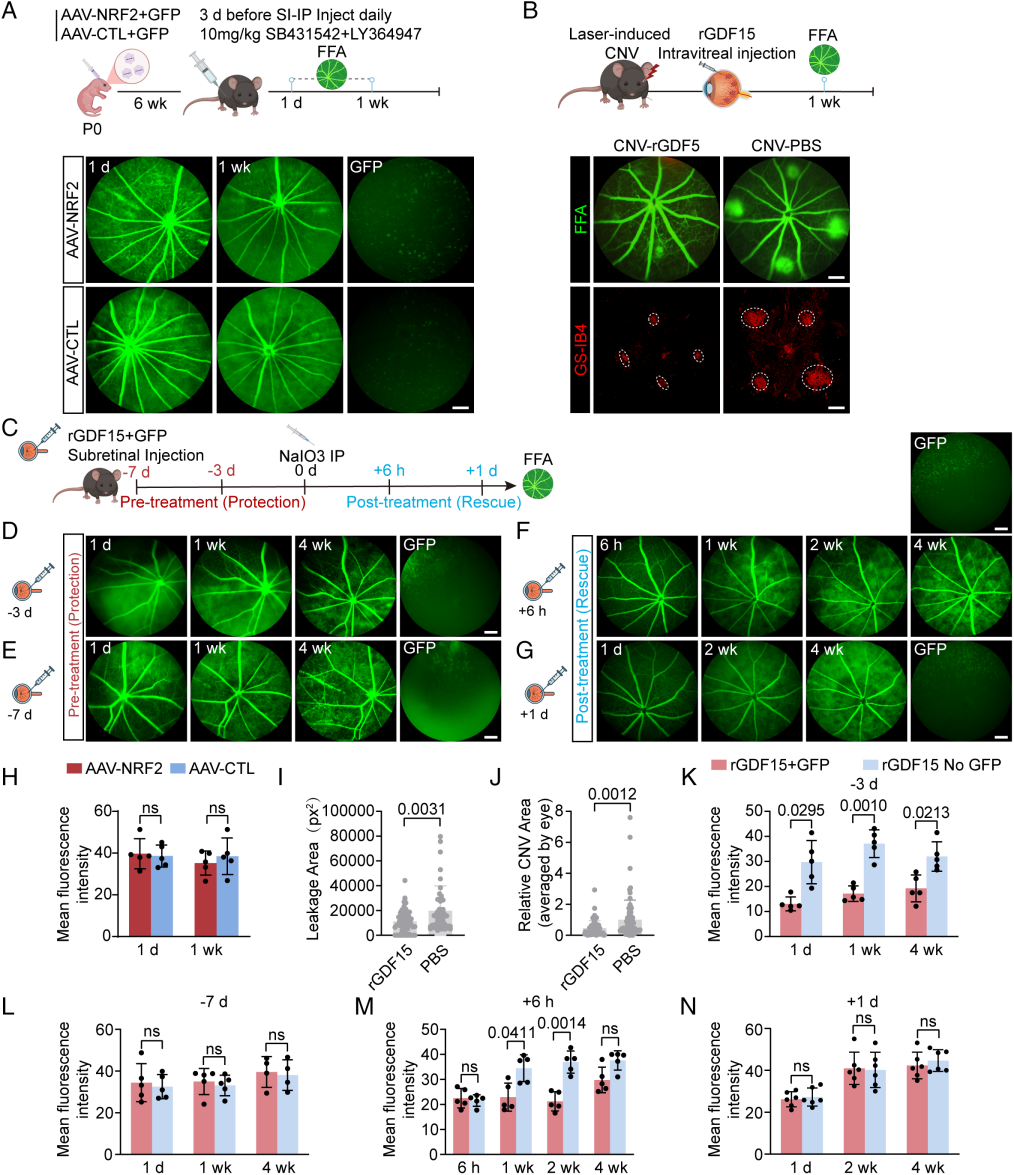
TGF-β pathway inhibition, choroidal neovascularization (CNV) testing, and temporal analysis of GDF15-mediated protection after NaIO_3_ injury. (*A*) Inhibitor study: P0 subretinal AAV8/BEST1-NRF2 or AAV-CTL with AAV-GFP, followed in adulthood by daily intraperitoneal SB431542 and LY364947 beginning 3 d before NaIO_3_. FFA images over time. (Scale bar, 100 μm.) (*B*) Experimental design for the laser-induced CNV model. FFA images and GS-IB4-stained CNV lesions. (Scale bar, 100 μm.) (*C*) Temporal analysis of rGDF15 protection or rescue. (*D* and *E*) Pretreatment: Mice received rGDF15 3 d (*D*), 7 d (*E*) before NaIO_3_. FFA images over time. (Scale bar, 100 μm.) (*F* and *G*) Posttreatment: Mice received rGDF15 6 h (*F*), 1 d (*G*) after NaIO_3_. FFA images over time. (Scale bar, 100 μm.) (*H*) Quantification of fluorescein intensity in *A* (n = 5 eyes per group, mean ± SD, two-way ANOVA with Šídák’s multiple-comparisons test). (*I*) Quantification of leakage area in *B*. (*J*) Relative CNV lesion area in *B* (n = 19 eyes per group for PBS, n = 17 eyes per group for rGDF15, mean ± SD, unpaired *t* test). (*K*–*N*) Quantification of mean fluorescein intensity in GFP-positive and GFP-negative regions shown in *D*–*G* (n = 5 to 6 eyes per group, mean ± SD, two-way ANOVA with Šídák’s multiple-comparisons test).

To examine whether NRF2-mediated protection involves TGF-β-related signaling, TGF-β receptor signaling was pharmacologically inhibited by two inhibitors, SB431542 and LY364947, before oxidative injury (Fig. 7*A*). WT mice received neonatal subretinal injection of AAV8/BEST1-NRF2 or AAV-CTL, followed by daily treatment with SB431542 and LY364947 beginning 3 d before NaIO_3_ administration in adulthood. FFA showed that when TGF-β receptor signaling was inhibited, eyes infected by AAV8/BEST1-NRF2 exhibited retinal hyperfluorescence after NaIO_3_ injury, comparable to that observed in AAV-CTL treated eyes (Fig. 7 *A* and *H*). To further evaluate TGF-β signaling, immunohistochemistry for pSMAD, the downstream effector of TGF-β receptor signaling, was performed. WT mice received subretinal rGDF15 or PBS injections and eyes were analyzed 24 h later (*SI Appendix*, Fig. S7). rGDF15-injected eyes exhibited increased pSMAD2/3 immunoreactivity in both retina and choroidal vascular cells relative to PBS-injected contralateral eyes. Pretreatment with SB431542 and LY364947 reduced this response. Very little pSMAD immunoreactivity was seen in the RPE or photoreceptors. These observations are consistent with activation of SMAD2/3 signaling following local GDF15 delivery.

### GDF15 Reduces CNV Lesion Size in a Laser-Induced CNV Model.

To determine whether the vascular protective effects of GDF15 extend beyond NaIO_3_-induced oxidative injury, rGDF15 was tested in a laser-induced CNV model (Fig. 7*B*). Intravitreal injection of rGDF15 was performed on the same day as laser injury, and FFA was carried out 7 d later to assess leakage of induced lesions. Representative FFA images and CNV flat mounts showed reduced hyperfluorescence and smaller CNV lesions in GDF15-treated eyes compared with controls (Fig. 7*B*). Quantification further confirmed that GDF15 treatment decreased CNV leakage and lesion size relative to controls (Fig. 7 *I* and *J*).

### GDF15 Exerts Protection Within an Early Temporal Window.

It was of interest to determine whether rGDF15 could confer protection when administered at different time points relative to oxidative injury (Fig. 7*C*). When rGDF15 was delivered 3 d before NaIO_3_ exposure, retinal vascular abnormalities were markedly reduced (Fig. 7 *D* and *K*). In contrast, when rGDF15 was administered 7 d before NaIO_3_ injury, no protective effect was observed (Fig. 7 *E* and *L*). Shorter post injury time points also were tested. Administration of rGDF15 6 h after NaIO_3_ injection partially reduced retinal hyperfluorescence (Fig. 7 *F* and *M*), whereas administration 24 h after injury showed minimal protective effect (Fig. 7 *G* and *N*). These findings indicate that GDF15 mediated protection occurs within a limited temporal window.

## Discussion

Oxidative stress is proposed to be a driver of dry AMD and related retinal disorders (1–3). The outer retina, RPE, Bruch’s membrane, and choriocapillaris function as a tightly coupled unit, so structural and molecular changes in one compartment may propagate to adjacent tissues (6–10). In the present study, systemic NaIO_3_ injury produced abnormalities in both retinal and choroidal vascular beds, with selective vulnerability of the DCP and marked disorganization and a reduced capillary density in the choriocapillaris. These changes were accompanied by substantial ERG deficits. Despite these acute and drastic changes, AAV8/BEST1-NRF2 was able to almost completely protect these tissues. The current results demonstrating the protection of vessels extends our earlier work showing that AAV8/BEST1-NRF2 overexpression preserved the RPE and photoreceptors, as well as vision, in the NaIO_3_ model as well as in models of retinitis pigmentosa (22, 23, 33).

A key finding is that NRF2-CM can transfer partial protection in vivo, supporting our hypothesis that RPE-specific NRF2 overexpression induces soluble protective factor(s). The ability of NRF2-CM to reduce abnormal angiographic signals, preserve deep plexus structure, and maintain choroidal vascular organization strongly supports a non-cell-autonomous mechanism mediated by soluble factors, rather than by action only within cells that directly transcribe NRF2 target genes. The spatial restriction of protection to regions exposed to NRF2-CM suggests a local paracrine effect rather than systemic effect. Our findings support a model in which NRF2 converts the stressed RPE from a source of injury propagation into a source of protective intercellular signaling. This finding suggests that RPE-centered therapies may protect vascular beds and neural tissue.

By integrating prior bulk RNA-seq data with single-cell receptor maps and validating the candidate at the protein and functional levels, we identified GDF15 as a downstream mediator of the NRF2-induced protection. GDF15 is a stress responsive cytokine with context-dependent effects on metabolism, inflammation, and tissue adaptation (25, 26). In the eye, GDF15 is induced after retinal injury, can protect retinal ganglion cells, and can modulate retinal inflammation in autoimmune uveitis models (29–31). In the NaIO_3_ injury model, rGDF15 recapitulated major features of the NRF2-protective phenotype, whereas genetic loss of Gdf15 partially weakened NRF2-mediated protection of vascular structure and retinal function. However, the phenotype was not completely eliminated in Gdf15-deficient mice, indicating that GDF15 should be regarded as one effector rather than the sole effector. NRF2 is a regulator of hundreds of genes and might be predicted to induce more than one protective factor (18).

The relationship between NRF2-CM and rGDF15 also supports the model that Gdf15 is not the only protective factor. Comparison of the amount of GDF15 in the CTL-CM and NRF2-CM revealed approximately a twofold difference, in keeping with the difference in the RNA-seq data (22). Dilution experiments with the CTL-CM and NRF2-CM revealed that there was more activity in the NRF2-CM than accounted for by GDF15 alone. In addition, the amount of GDF15 measured in the NRF2-CM was much lower than that required for protection by rGDF15. One interpretation is cooperativity: GDF15 may act together with other NRF2-induced soluble factors in the CM, thereby amplifying protection beyond what rGDF15 can achieve by itself. A second, not mutually exclusive, explanation is that endogenous GDF15 induced by NRF2 differs from rGDF15 in some way so as to increase its activity, perhaps by modifications that enhance its specific activity or stability. Prior work in other systems has shown that endogenous GDF15 can participate in anti-inflammatory programs downstream of NRF2 activation e.g., refs. 34 and 35. Interestingly, rGDF15 was not able to recapitulate the effects predicted by GDF15 induction in hepatocytes by NRF2, leading to the suggestion that endogenous GDF15 may be more active due to modifications not made on recombinant forms (35).

The effects of GDF15 in this study were not restricted to vascular morphology. rGDF15 also preserved the RPE and sustained more photoreceptors than the control after injury. It is not clear if the effects of rGDF15 are via one particular cell type, with indirect effects on others, or whether more than one cell type directly responds to the addition of rGDF15. One interesting aspect of the effects of GDF15 on the RPE concerns its sufficiency vs. necessity. Addition of rGDF15 was sufficient to protect the RPE. However, AAV8/BEST1-NRF2 still protected the RPE in the Gdf15^−/^^−^ mice. Protection of the RPE in the absence of GDF15 may not be surprising given that NRF2 induces many more genes than GDF15 in the RPE (22), likely at least providing protection from oxidative damage. The results from staining for pSMAD2/3 offer one explanation for these findings. Most of the pSMAD2/3 staining following GDF15 injection was in the choroidal vasculature. This might suggest that protection of the RPE was due to GDF15’s protection of the choroid. Given that NaIO_3_ is delivered systemically, vessels might be the first cell type to experience NaIO_3_-induced injury, which then affects the nearby RPE. Photoreceptor damage might follow RPE damage, and/or damage to retinal vessels, once the NaIO_3_-induced treatment has damaged them.

The protection of vessels by either NRF2 overexpression in the RPE, or by addition of GDF15, can be considered in light of AMD pathology. Wet AMD occurs when choroidal vessels invade the retina, perhaps in response to inadequate trophic support from an ailing RPE (36). By improving the health of the RPE, potentially in dry AMD, the probability of conversion of dry to wet AMD could be reduced. Similarly, the fairly broad protection of vessels, RPE and photoreceptors may predict a therapeutic effect of NRF2 overexpression in the RPE, or addition of GDF15, for diabetic retinopathy where vessels are affected (5).

The receptor landscape for GDF15 in peripheral tissues remains unresolved. While circulating GDF15 signals through the hindbrain GFRAL receptor, which recruits RET for its effects (27, 28), GFRAL expression has been documented to be absent in peripheral tissues and outside of the brainstem in the brain (37). Our observation of increased pSMAD2/3 immunoreactivity in ocular tissues following GDF15 injection, with attenuation by SB431542 and LY364947, supports signaling via the TGF-β receptor. SMAD phosphorylation following addition of GDF15 to retinal cultures in other studies offers additional evidence for this signaling pathway (38). However, additional work will be needed to establish TGF-β receptor as the relevant receptor. It is also worth noting that the rGDF15 used here was produced in *Escherichia coli*, making contamination by mammalian TGF-β family proteins during production a less likely explanation for the observed activity (39).

The laser-induced CNV model is widely used as an experimental surrogate for neovascular AMD (40, 41). When rGDF15 was delivered intravitreally on the day of laser injury, the injured eyes showed reduced fluorescein leakage at day 7, together with smaller CNV lesions. Additionally, the protection of ganglion cells, amelioration of uveitis, as well as the activities of GDF15 in other disease models, suggest that GDF15 might not only be a marker of stress, but can exert protective effects more broadly (29, 30).

Finally, the temporal dependence of protection is one of the more clinically informative aspects of this work. Protection was evident when rGDF15 was delivered 2 to 3 d before injury and was still effective when administered 6 h after NaIO_3_. However, protection was lost with a 7 d pretreatment interval or a 1 d post injury delay. Oxidative injury likely initiates rapidly, but the downstream collapse of vascular stability, barrier integrity, and inflammatory homeostasis likely unfolds over time. Once that cascade progresses beyond a threshold, later intervention becomes ineffective. This interpretation is consistent with broader work in oxidative retinal degeneration showing that timing strongly constrains rescue efficacy (3, 13).

## Materials and Methods

### Animals.

Adult C57BL/6J mice were used for WT experiments. Gdf15 knockout mice were obtained from Randy Seeley and maintained as homozygous breeding pairs (42). Both male and female mice were included. All procedures were approved by the Harvard University Institutional Animal Care and Use Committee.

### AAV Vectors and Subretinal Delivery.

AAV8/BEST1-NRF2 was used to express human NRF2 in RPE cells. AAV-CTL served as the control vector, and AAV-GFP was coadministered to mark transduced regions (23). Vectors were produced in HEK293T cells and purified by iodixanol gradient ultracentrifugation (33).

P0 pups received subretinal AAV8/BEST1-NRF2 or AAV-CTL at 4 × 10^^^8 vector genomes (vg)/eye plus AAV-GFP tracer at 1 × 10^7 vg/eye (23). Approximately 0.25 μL was delivered per eye. One eye received AAV8/BEST1-NRF2 and the contralateral eye received AAV-CTL, with laterality alternated across litters. Animals with less than 80% posterior RPE transduction were excluded. For adult injections, 6 to 8-wk-old mice received approximately 1 μL subretinal viral suspension. AAV8/BEST1-NRF2 or AAV-CTL was administered at 1 × 10^9 vg/eye with AAV-GFP tracer at 1 × 10^7 vg/eye. Transduction was limited to the injection bleb region.

### Conditioned Medium Preparation, Administration, and ELISA.

For CM experiments, mice received P0 subretinal AAV8/BEST1-NRF2 or AAV-CTL injections. At 8 wk, RPE–choroid complexes were cultured RPE side up for 24 h in serum-free explant medium with three explants per 500 μL. For subretinal administration, centrifuged media were separated using 10-kDa molecular-weight cutoff filters, and adult recipient mice received 1 μL of NRF2-CM or CTL-CM fraction with GFP tracer (1 × 10^7 vg/eye), followed by NaIO_3_ injury 2 d later. For dilution experiments, centrifuged media were diluted 1:10 before injection. GDF15 levels were measured using uncentrifuged CM with the Mouse/Rat GDF-15 Quantikine ELISA Kit.

### Recombinant Protein Administration.

Recombinant mouse GDF15 was administered subretinally to adult mice, typically 2 d before NaIO_3_ injury. rGDF15 was delivered at 10 μM in 1 μL per eye (31), with dose–response experiments using 50, 10, or 1 ng per eye. For timing experiments, rGDF15 was injected 7, 3, or 2 d before, or 6 h or 1 d after, NaIO_3_ injury. The contralateral eye received PBS. Recombinant mouse oncostatin M was delivered subretinally at 100 ng per eye (43).

### NaIO_3_ Injury and CNV Model.

NaIO_3_ was freshly dissolved in sterile saline and administered intraperitoneally at 75 mg/kg. Control mice received saline. For laser-induced CNV, four laser burns were applied per eye using the Micron IV image-guided system with a 532-nm laser (44). Burns were placed equidistant from the optic nerve, and only lesions with a vaporization bubble were included. Immediately after laser injury, mice received intravitreal rGDF15 or PBS. At 7 d, FFA was performed and eyes were collected for flat-mount analysis.

### FFA and ERG.

FFA was performed using the Micron IV system. After intravenous injection of 1% sodium fluorescein, images were acquired 1 min later. In the NaIO_3_ model, angiographic hyperfluorescence was quantified in ImageJ by measuring extravascular fluorescence while excluding major retinal vessels. For adult partial-transduction experiments, analysis was restricted to GFP-positive regions. For CNV, individual lesions were manually outlined and hyperfluorescent lesion areas were quantified.

Ganzfeld ERG was performed using an Espion E3 system. Mice were dark-adapted for at least 2 h before recording. Scotopic responses, oscillatory potentials, and photopic responses after light adaptation were collected and analyzed using Espion software (23).

### Pharmacological Inhibition.

To inhibit TGF-β receptor signaling, mice received daily intraperitoneal SB431542 and LY364947 at 10 mg/kg each, beginning 3 d before NaIO_3_ injection and continuing until 7 d after injury (45).

### Tissue Processing, Staining, and Imaging.

Eyes were dissected into neural retina and RPE–choroid complexes. Retinas were stained with GS-IB4 to label retinal vasculature or with anti-cone arrestin antibody for cone analysis. RPE flat-mounts were stained with phalloidin to visualize RPE cell morphology. For pSMAD2/3 analysis, cryosections were stained with anti-pSMAD2/3 antibody and GS-IB4. For choroidal vascular staining, RPE–choroid tissues were treated with EDTA to remove RPE, bleached with hydrogen peroxide, and stained with anti-podocalyxin antibody (46). Samples were mounted as flat-mounts and imaged using an Olympus VS200 slide scanner or fluorescence imaging systems.

### Image Analysis.

Image analysis was performed using ImageJ/Fiji and REAVER. For RPE quantification, four regions were sampled from each flat-mount, avoiding damaged or out-of-focus areas, and RPE cells were manually counted (23). For adult partial-transduction experiments, GFP-positive and GFP-negative regions were analyzed. Retinal vascular parameters were quantified using REAVER (47), including vessel area fraction, vessel length, segment count, and branchpoint number. Cone numbers were counted from representative regions.

### Statistics.

Statistical analyses were performed using GraphPad Prism and Microsoft Excel. Unless stated, n represents individual eyes. Data are presented as mean ± SD. Paired or unpaired two-tailed Student’s *t* tests, nonparametric tests, one-way ANOVA, or two-way ANOVA with multiple-comparisons correction were used as appropriate. Contralateral-eye comparisons were analyzed as paired samples, and GFP-positive vs. GFP-negative regions within the same eye were analyzed using eye-level paired summary values.

## Supplementary Material

Appendix 01 (PDF)

## Data Availability

Study data are included in the article and/or *SI Appendix*.
